# 

**Published:** 1838-03-28

**Authors:** 


**TERMS.**

The Examiner is published every alternate Wednesday, at *Three dollars* per annum, *payable in every instance in advance.* Two copies, if sent to the same address, for Five dollars.

Persons disposed to subscribe, will please send their address by mail, *accompanied with a remittance.*

Letters and communications may be addressed to the "Editors of the Medical Examiner," and must be *post paid, otherwise they will not be taken from the office.*

No subscriptions received for less than one year, commencing January 3d, 1838.

Advertisements inserted at the rate of $1 for twelve lines. OfficeNo. 230 Spruce street, between Sixth and Seventh sts. South side.

